# Predicting the Rate Structure of an Evolved Metabolic Network

**DOI:** 10.3390/metabo15030200

**Published:** 2025-03-13

**Authors:** Friedrich Srienc, John Barrett

**Affiliations:** Department of Chemical Engineering and Materials Science and BioTechnology Institute, University of Minnesota, Minneapolis/St. Paul, MN 55455/55108, USA; barre158@umn.edu

**Keywords:** metabolic networks, elementary flux modes, evolution, statistical thermodynamics

## Abstract

*Background*: When glucose molecules are metabolized by a biological cell, the molecules are constrained to flow along distinct reaction trajectories, which are defined by the cell’s underlying metabolic network. *Methods*: Using the computational technique of Elementary Mode Analysis, the entire set of all possible trajectories can be enumerated, effectively allowing metabolism to be viewed in a discretized space. *Results*: With the resulting set of Elementary Flux Modes (EMs), macroscopic fluxes, (of both mass and energy) that cross the cell envelope can be computed by a simple, linear combination of the individual EM trajectories. The challenge in this approach is that the usage probability of each EM is unknown. But, because the analytical framework we have adopted allows metabolism to be viewed in a discrete space, we can use the mathematics of statistical thermodynamics to derive the usage probabilities when the system entropy is maximized. The resulting probabilities, which obey a Boltzmann-type distribution, predict a rate structure for the metabolic network that is in remarkable agreement with experimentally measured rates of adaptively evolved *E. coli* strains. *Conclusions*: Thus, in principle, the intracellular dynamic properties of such bacteria can be predicted, using only the knowledge of the DNA sequence, to reconstruct the metabolic reaction network, and the measurement of the specific glucose uptake rate.

## 1. Introduction

**Chemical and entropic forces**

One of the great challenges in understanding the living world is predicting how the set of observable characteristics (phenotype) arises from the genetic makeup of the individual in the context of a given environment [1]. This is facilitated by the development of new research tools that have revolutionized our ability to investigate and manipulate the genome, the cellular composition and to measure multiple aspects of the environment [2,3,4]. In biological systems research, the task is now to assimilate this information into causal, predictive relationships. The connection between the genome and the phenotype of an organism in response to the environment is mediated by the reactions that convert the information present in the genome into the structure and functioning of an organism. Thus, understanding metabolic networks and how they evolve is expected to profoundly contribute to solving the grand challenge in systems biology.

Biological systems, like any other system in the universe, must obey the fundamental principles of thermodynamics. Thus, the organization, functioning and evolution of life, as mediated by the reaction network of cells, must be explainable at the level of these fundamental rules.

In physics, entropic forces are attributed to the tendency of matter to bring a *physical* system, through the effects of thermal fluctuations, toward a macroscopic state in which the number of microscopic states (or micro-states) that are compatible with this macroscopic state is maximized. The average of all the microscopic states reflects the measured properties of the macroscopic states. In other words, thermal fluctuations tend to bring a system toward its macroscopic state of maximum entropy [5,6]. Similarly, in *biological* systems involving cells that must constantly evolve to survive, one can argue that entropic forces are also at work, based on the effects of genetic fluctuations, to bring the system toward a macroscopic state of maximum entropy. Thus, cells growing in a given environment are subject to both chemical and entropic forces. Chemical forces, due to the imbalance of chemical potentials between substrates and products, drive the metabolic reactions, while entropic forces drive the evolution of the metabolic network toward a state that maximizes the entropy of the system in which the cells grow.

Here, we develop the thermodynamic relationships that support these statements. They provide predictions for the structure of a metabolic network that can be validated by comparison with experimental measurements. It is well established that in isolated systems at steady state, the chemical potentials are equilibrated such that the Gibbs free energy is minimized, and the entropy is maximized. Both the Gibbs free energy of reaction and entropy of reaction become zero when the system reaches a time invariant state in equilibrium [7]. While these principles are firmly grounded for equilibrium systems, there is increasing evidence that they can be extended also to open, non-equilibrium systems that exchange material and energy with the environment [8]. Open, non-equilibrium systems can also reach a time invariant state when they are at steady state. We will assert that open systems in such a state also exhibit extrema in the considered thermodynamic properties. The Gibbs free energy tends towards a minimum as the chemical potentials drive the reactions into the dynamic steady state of reactant concentrations [9], while the associated entropy of the system represents a maximum with respect to the organization of the cell’s metabolic network and the surrounding environment.

Here, we apply the Maximum Entropy Production (MEP) principle as it relates generally to a reacting system in a dynamic steady state. MEP has been attributed as a fundamental physical principle in many phenomena including, for example, the Earth’s climate system (e.g., [10,11]; thermal convection [12]; electrical currents [13,14]; crystalline solids [15]; ecological systems [16] and biochemical processes [17,18]. A similar derivation has been developed before based on a control volume approach and a dimensionless potential function that is a minimum when the entropy production rate is at a maximum [9,19].

As a mathematical basis for our model, we employ the computational technique of Elementary Mode Analysis (EMA). The value of EMA and other techniques for metabolic modeling has increased in recent years given the extensive progress that has been made in the reconstruction and understanding of metabolic networks based on detailed genomic information and experimental techniques for quantifying flux values. A metabolic network can function according to many different pathway options. Such redundancy of pathways enables cells to compete efficiently and to survive under changing environmental conditions [20]. Elementary mode (EM) analysis has emerged as a powerful systems biological tool that rigorously dissects a metabolic network into its basic building blocks [21,22]. Consequently, the use of this approach allows the metabolism of a functioning cell to be viewed as a weighted average of the fluxes through all fundamental pathways (EMs) supported by the metabolic network [23,24]. The set of EMs represents the parts list for cell function encoded at a higher level in the hierarchy of biological complexity. Because each EM constitutes a balanced stoichiometric equation, this information can be used to estimate the thermodynamic properties of each EM, e.g., the Gibbs free energy of reaction ($∆G_{R}$) and the entropy of reaction ($∆S_{R}$).

An ideal system for investigating the fate of growing cells is the continuous stirred tank reactor (CSTR). It represents an open, non-equilibrium system in which the state of cells together with the growth environment are precisely defined when a steady state is reached. Therefore, such a system enables a quantitative assessment of how cells function based on the underlying metabolism and how they interact with their environment. Here, the quantitative macroscopic relationships are developed for the steady state of the system in which the chemical driving forces are minimized. By dissecting the metabolic network into its fundamental parts or elementary modes, metabolism can be discretized at the microscopic level. With this approach, the metabolic network becomes accessible to the rules of statistical thermodynamics that reveal the entropic forces that are at work in the evolving cell system. Analogous to the way in which statistical mechanical models must be informed by empirically measured properties of the macroscopic state, our model makes use of experimental measurements of the cell’s overall growth stoichiometry as well as environmental properties of the CSTR. Together, this information can be combined to estimate the rate of entropy production by the macroscopic, biological system. Finally, a probability distribution for the underlying microstates can be constructed whose mean matches the experimentally measured macroscopic state.

The developed theory enables the prediction of the internal fluxes through the metabolism of cells and their interactions with the growth environment. The predictions are in remarkable agreement with detailed experimental measurements of intracellular metabolic fluxes that have been recently made possible.

## 2. Results

### 2.1. Theory

We have previously attempted to relate the dynamic properties of a metabolic network to the inherent thermodynamic properties of such system.

Specifically, in Srienc and Unrean (2010) [25] we provide a derivation for the Boltzmann distribution of reaction entropies when the rate of type 2 entropy production rate (defined below) is maximized. In Unrean and Srienc (2011) [26] the entropy balance is derived and the data are then interpreted based on the previously presented statistical thermodynamical approach. Unfortunately, the derivations provided in these two papers are incomplete and as such not as transparent and comprehensive as one would expect from a self-consistent theory. Thus, in the current work these shortcomings have been eliminated and a consistent and complete theory is presented.

This has been accomplished by following novel aspects of the presented theory: (i) the affinity of reaction is introduced which provides the commonly accepted link to the rate of entropy production of a reacting system. We define this rate as the Type 1 entropy production rate; (ii) the results are expressed as the entropy and as the Gibbs free energy of the SYSTEM at steady state which has not been done before. It is shown that, at steady state, the minimization of the Gibbs free energy of the system corresponds to the maximization of the Type 1 entropy production rate which corroborates the MEP (Maximum Entropy Production) principle. Thus, the MEP principle is not assumed but derived from basic balances. In contrast, the entropy of the system is maximized when the Type 2 entropy production rate is maximized. The Type 2 entropy production rate lacks the enthalpic contribution present in the Type 1 rate. (iii) The statistical treatment introduces a new parameter of the system: the maximum attainable specific growth rate of the evolving system. This reveals the Boltzmann constant in the Boltzmann factor and unifies the data of the experimentally tested strains since the data collapse into one general relationship that is valid for all strains.

**Non-equilibrium Thermodynamics of open systems**

The entropy balance for a continuous stirred tank reactor (CSTR), representing a non-equilibrium, open system, results in the following expression (see Supplemental File S1 for a detailed derivation):

(1) $$\frac{dS}{dt}=\underset{i}{\sum }(s_{i,\;in}{\dot{n}}_{i,in}-s_{i}{\dot{n}}_{i})-\dot{\frac{Q}{T}}+{\dot{S}}_{gen}$$

Here, s_i,in_ (s_i_) are the molar entropies of the individual components [kJ/K/mol], at the corresponding concentrations, transported in (out) of the system at molar flow rates n_i,in_ (n_i_) [mol/h]; $\dot{Q}$ is the rate of heat transfer through the reactor walls [kJ/h], and ${\dot{S}}_{gen}$ [kJ/K/h] is the rate of internal entropy generation of the system due to the irreversibility of the process. The first two terms on the right-hand side represent the net entropy transported to the surroundings due to material transport and due to heat transfer, respectively. From this expression one can see that in a steady state situation with zero entropy accumulation, the internal entropy production term must be balanced by the transport of entropy to the surroundings. Due to the Second Law, the internal entropy production term must always be larger or equal to zero.

Using an energy balance to obtain $\dot{Q}$ and considering the irreversibilities in the system due to reaction and mixing, we can convert this expression at steady state into Equation (2) by substituting the molar flow rates ǹ_i_ with the product of volumetric flow rates F [L/h] and concentrations c_i_ [mol/L] and by introducing the space time τ = V/F [h]. The rate of entropy generation is given by the product of the entropy of reaction *ΔS_R_* and the extent of reaction $\dot{\xi }$ [mol/L.h] (see also Equation (11)). Recalling that entropy is an *extensive* property, we see that the expression provides a statement of the entropy content of the system at steady state.

(2) $$S_{sys}=\underset{i}{\sum }s_{i}c_{i}=\underset{i}{\sum }s_{i}c_{i,\;in}+\tau \dot{\xi }\Delta S_{R}$$

This expression shows that the system entropy is a function of the concentration of the species in the inlet stream, the entropy of the species evaluated at the system (outlet) conditions—this includes effects from dilution, temperature, pH, and ionic strength—and the rate of entropy production in the system contributed by the reaction entropy. Equation (2) shows that the system entropy is expected to increase as the rate of entropy production by reaction increases. Furthermore, when the system is operated at isothermal conditions, and dilution of the incoming stream is negligible, as is commonly the case for low cell-density, chemostat cultures, the reaction term dominates, and system entropy approaches a maximum. We define this entropy production rate as the Type 2 entropy production rate since it omits the enthalpic contribution to the entropy formation.

Using the Gibbs relation (*G*_sys_ = *H_sys_ − TS_sys_* ) along with expressions for *H_sys_* and *G*_sys_ that are derived similarly to the procedure used for Equation (2), we can derive an expression for the Gibbs free energy of the system:

(3) $$G_{sys}=H_{sys}-\underset{i}{\sum }h_{i}c_{i,\;in}-\tau \dot{\xi }\Delta H_{R}+\underset{i}{\sum }g_{i}c_{i,\;in}-T\tau \dot{\xi }\Delta G_{R}$$

By combining Equation (2) with different statements of the Gibbs relation (*G*_sys_ = *H_sys_* − *TS_sys_*, *g_i_ = h_i_ − Ts_i_*, and Δ*G_R_ = ΔH_R_ − TΔS_R_*), we can convert this to the following expression for the Gibbs free energy of the system given that the first three terms on the right hand side of Equation (3) must sum to zero

(4) $$G_{sys}=\underset{i}{\sum }g_{i}c_{i,\;in}+\tau \dot{\xi }\Delta G_{R}$$

The rate of entropy production due to the irreversibility of the reaction can also be expressed as

(5) $$V{\dot{\sigma }}_{rxn}={\dot{S}}_{rxn}=V\frac{A}{T}\dot{\xi }$$

where ${\dot{\sigma }}_{rxn}$ [J/K.L.h] is the rate of entropy production per unit volume, and *A* [J/mol] is the affinity of reaction defined as the negative of the Gibbs free energy of reaction [27]:

(6) $$A\equiv -\Delta G_{R}$$

With these relations, we can write

(7) $$G_{sys}=\underset{i}{\sum }g_{i}c_{i,\;in}-\tau T{\dot{\sigma }}_{rxn}$$

The Gibbs free energy of the system at steady state is proportional to the negative rate of entropy production ${\dot{\sigma }}_{rxn}$. We define this entropy production rate as the Type 1 production rate since it expresses the commonly known entropy production rate based on the affinity of reaction that includes contributions from both the enthalpy as well as entropy of reaction. Thus, a reacting open system adjusts the component concentrations because of reactions such that the Gibbs free energy at steady state is at a minimum due to the tendency to equilibrate chemical potentials. This is accomplished when the rate of entropy production is maximized corroborating the Maximum Entropy Production (MEP) principle [28,29]. But the corresponding system entropy (Equation (2)), under the assumed experimental conditions, depends only on the rate of entropy formation due to the entropy of reaction. From the contributions to the internal entropy generation expression, only the reaction entropy affects the entropy of the system, because the enthalpic component of the internal entropy generation is exported into the surroundings and does not contribute to the entropy content of the system. The obtained relationships describe the macroscopic behavior of the system and are generally valid for any reacting, non-equilibrium system at steady state. A detailed derivation including material and energy balances is given in Supplemental File S1.

**Thermodynamics of Elementary Modes**. An Elementary Mode (EM) is formally defined as a minimal set of enzymes that can operate at steady state with all irreversible reactions proceeding in the appropriate direction [30]. An alternate definition that can be easier visualized, is that an EM represents a reaction sequence (or pathway) that a glucose molecule follows when it is metabolized. At the dynamic steady state, the overall growth reaction can be formally represented by the general chemical equation

(8) $$A+\;\nu_{B}B+\ldots =\nu_{C}C+\nu_{D}D+\ldots \;\;\;$$

that represents all components (nutrients, biomass and products) in the reactor. Here, one mole of glucose (A) plus nutrients (B) get converted into biomass (C) and products (D). The factors ν_ι_ represent the individual stoichiometry coefficients or the molar yields per one mole of glucose utilized of each component. They are negative for reactants and positive for products. The rate of reaction is described by the extent of reaction $\dot{\xi }$ [mol/L.h]. The extent of reaction represents also the rate of glucose consumption since the stoichiometry coefficient for glucose is −1. The rate of the chemical growth reaction (Equation (8)) is proportional to the biomass concentration or the number of cells present. Because the reaction equation holds for all biomass concentrations it is convenient to express it on a per cell mass basis. The rates of reaction then become specific rates of reaction $\hat{\xi }$ [mol/h.g CDW].

Such a chemical equation can be written for each EM. It involves only the external metabolites that are taken up or that are excreted by the cells. Each EM contributes to the specific glucose uptake at a rate defined by

(9) $$\;{\hat{\xi }}_{j}=\;\hat{\xi }\;p_{j}$$

where p_j_ represents the fraction of the total specific glucose uptake rate $\hat{\xi }$ [mol/h.g CDW] that is consumed by elementary mode ‘j’. Thus, each elementary mode contributes to the overall metabolism with a usage probability p_j_.

For each EM (*j*), the entropy, Gibbs free energy and enthalpy of reaction can be computed based on the individual, molar properties of formation (i) summed over the *m* reactants participating in the growth equation (see Equation (8))

(10) $$\Delta s_{r,j}=\overset{m}{\underset{i}{\sum }}\nu_{i,j}s_{i}\;\Delta g_{r,j}=\overset{m}{\underset{i}{\sum }}\nu_{i,j}g_{i}\;\Delta h_{r,j}=\overset{m}{\underset{i}{\sum }}\nu_{i,j}h_{i}$$

Here, we use a lowercase (Δ*s_r,i_,* Δ*g_r,i_,* or Δ*h_r,j_*) to represent the reaction properties of a single EM. However, the same relation is also true, for the overall chemical reaction of the cell. Thus, the macroscopic *rate* of entropy, Gibbs free energy and enthalpy generation ($\dot{S}\;$,$\;\dot{G},\;\dot{H})$ can be defined in terms of the individual EM reaction properties

(11) $$\dot{S}=\hat{\xi }\;\Delta S_{R}=\overset{n}{\underset{j}{\sum }}{\hat{\xi }}_{j}\Delta s_{j}\;\dot{G}=\hat{\xi }\;\Delta G_{R}=\overset{n}{\underset{j}{\sum }}{\hat{\xi }}_{j}\Delta g_{j}\;\dot{H}=\hat{\xi }\;\Delta H_{R}=\overset{n}{\underset{j}{\sum }}{\hat{\xi }}_{j}\Delta h_{j}$$

where $\hat{\xi }$ [moles glucose/h.g CDW] is the cell specific glucose consumption rate in the reactor, ∆S_R_ [J/K.mole glucose] is the reaction entropy of the overall growth reaction per mole glucose consumed and ∆G_R_ and ∆H_R_ the corresponding Gibbs free energy and enthalpy of reaction [J/mole glucose]. $\dot{S}$ [J/K.h.g CDW] is the cell specific rate of entropy production, $\dot{G}$ and $\dot{H}$ [J/h.g CDW] are the cell specific rate of Gibbs free energy and enthalpy production, ${\hat{\xi }}_{j}$ [mole glucose/h.g CDW] is the specific glucose uptake reaction rate of elementary mode j and ∆s_j_, ∆g_j_, ∆h_j_ and p_j_ are the respective reaction properties and usage probabilities of individual elementary modes, respectively. Note that for simplicity the subscript r is omitted for the reaction properties of individual elementary modes, and the thermodynamic properties of the *overall* cell reaction are assigned using uppercase letters ($\Delta S_{R},\;\Delta G_{R},\;\Delta H_{R}$). After substituting with Equation (9) one obtains

(12) $$\dot{S}=\hat{\xi }\;\overset{n}{\underset{j}{\sum }}p_{j}\Delta s_{j}\;\dot{G}=\hat{\xi }\;\overset{n}{\underset{j}{\sum }}p_{j}\Delta g_{j}\;\dot{H}=\hat{\xi }\;\overset{n}{\underset{j}{\sum }}p_{j}\Delta h_{j}$$

Equation (12) shows that the cell specific rate of production of the thermodynamic reaction properties is a function of the usage probability of individual elementary modes and of the total glucose uptake rate of a cell. In fact, once the usage probabilities of elementary modes are known the specific rates of change relative to the glucose uptake rate are defined by the overall stoichiometry of the growth reaction (Equation (8)) since the stoichiometry coefficients (or yields) can be computed from the sum of contributions of individual elementary modes.

(13) $$\;\nu_{i}=\;\overset{n}{\underset{j}{\sum }}\nu_{j,i}p_{j}$$

where ν_i_ is the stoichiometry coefficient of the ith reactant in the overall growth equation and ν_j,i_ is the corresponding stoichiometry coefficient in the elementary mode j.

The specific rate of Gibbs free energy production (Equation (12)) can be combined with Equations (5) and (6) to obtain the specific rate of entropy production of the system due to the irreversibility of the reaction system:

(14) $${\dot{\sigma }}_{rxn}=\overset{n}{\underset{j}{\sum }}{\dot{\sigma }}_{rxn,j}=\overset{n}{\underset{j}{\sum }}{\hat{\xi }}_{j}\left(\frac{A_{j}}{T}\right)=\overset{n}{\underset{j}{\sum }}p_{j}\left(\frac{\hat{\xi }{\;A}_{j}}{T}\right)=\overset{n}{\underset{j}{\sum }}p_{j}\left(\frac{-\Delta g_{j}\hat{\xi }}{T}\right)\;$$

The corresponding entropy production rate due to individual reaction entropies becomes

(15) $$\dot{S}=\overset{n}{\underset{j}{\sum }}{\dot{S}}_{j}=\overset{n}{\underset{j}{\sum }}p_{j}\left(\Delta s_{j}\hat{\xi }\right)\;$$

It is important to distinguish between the two rates of entropy production defined by Equations (14) and (15). In the first case (Equation (14)), designated as type 1, the total entropy production rate reflects the irreversibility of reactions (entropy of reaction and entropy due to heat generation). In the second case (Equation (15)), designated as type 2, the entropy production rate reflects the contribution by the entropy of reaction *only* (type 2). The previously derived macroscopic relations have shown that the type 1 entropy production rate is a maximum when the Gibbs free energy of the system is a minimum at steady state (see Equation (7)). In contrast, the type 2 entropy production rate is a maximum when the entropy of the system is at a maximum (see Equation (2)). Clearly, both types of entropy production rates are defined by the internal rate structure of the metabolism, i.e., by the thermodynamic properties of external substrates, by the usage probability of each elementary mode and by the specific rate of glucose uptake.

Thus, the question arises whether the internal elementary mode structure of a cell evolves to minimize the Gibbs free energy of the system or to maximize the entropy of the system. In the first case the Gibbs free energies of reaction are the characteristic properties of the reaction trajectories defined by individual elementary modes as they determine the type 1 entropy production rate. In the second case, the entropies of reaction define the rate structure of the metabolism based on the type 2 entropy production rates of individual elementary modes.

**Frequency Distribution of Elementary Modes**

Having derived the equations that link a cell’s macroscopic rate of entropy production to the EM microstates of its metabolic network, the challenge then becomes to adjust the individual usage probabilities (*p_j_*) so that the rate of entropy production by the system is a maximum. At the same time, the probability distribution must be made to satisfy three constraints: (i) the fair apportionment of outcomes, (ii) a constant macroscopic specific entropy production rate, and (iii) unity of the sum of all probabilities [31]. The solution to this maximization problem, which is obtained by the method of Lagrange multipliers, represents then the constrained maximum specific entropy production rate with respect to the underlying variation in usage probabilities of elementary modes. This approach has been previously described for the case of maximizing the entropy of the system on the basis of maximizing the type 2 entropy production rate [25].

Alternately, one can carry out the thought experiment as performed originally by Boltzmann [32] for the energy distribution in gas particles. But instead of observing the *energy content* of individual particles, one observes the *time trajectories* of individual glucose molecules when they are metabolized, and one evaluates the associated rate of specific entropy production. One should recall that individual glucose molecules are always metabolized following a path along an elementary mode. Arranging the same number of glucose molecule trajectories in all possible permutations yielding the fixed macroscopic specific entropy production rate, results in the most probable distribution of the usage of individual elementary modes.

Both approaches result in following expressions for the usage frequency of an elementary mode which represents a constrained maximum of the overall entropy production rate depending on which type of entropy production rate (type 1 or 2) determines the distribution:

(16) $$p_{j}=\exp(-\hat{\xi }∆s_{j}K+c)$$

or

(17) $$p_{j}=\exp(-\frac{\hat{\xi }\left(-∆\;g_{j}\right)}{TK}+c)$$

In linearized form the equations become

(18) $$\ln p_{j}=-\frac{\hat{\xi }∆s_{j}}{K}+c$$

or

(19) $$\ln p_{j}=-\frac{\hat{\xi }\left(-∆\;g_{j}\right)}{TK}+c$$

This expression relates the usage probability of an elementary mode j to the net glucose uptake rate of the cell $\hat{\xi }$ [moles glucose/h.g CDW] and to the individual entropies of reaction $∆s_{j}$ or Gibbs free energies of reaction $∆\;g_{j}$ of elementary modes. K and c are the Lagrange multipliers arising via the constrained optimization.

To keep dimensional consistency, we can separate from *K* the constant Q = 1 [1/h.g CDW] to give

(20) K=QR

with R [J/K.mol] representing the universal gas constant (or *molar* Boltzmann constant).

The usage probability of elementary modes based on type 2 entropy production rates becomes

(21) $$\ln p_{j}=-\frac{\hat{\xi }∆s_{j}}{Q\;R}+c$$

To obtain the constant c Equation (16) can be rewritten as

(22) $$p_{j}=exp^{c}\exp(-\frac{\hat{\xi }∆s_{j}}{QR})$$

Then, by summing up all probabilities to unity, one obtains the “partition” function Z along with the value of *C*

(23) $$exp^{-c}=Z=\overset{n}{\underset{j}{\sum }}\exp\left(-\frac{\hat{\xi }∆s_{j}}{QR}\right)$$

The unique form of Equations (16) and (17), suggests that the evolution of a metabolic network involves an interplay between two mechanisms, each having the ability to advance the fitness of the cell. The first mechanism is due to changing the network structure as reflected in the distribution of usage probabilities p_j_ of elementary modes. The second mechanism is due to the selection process reflected by the specific glucose uptake rate $\hat{\xi }$. The network structure determines the yield of biomass on glucose (*Y*) (see Equation (13)), and when this is multiplied by the specific glucose uptake rate, the resulting value gives the specific growth rate,

(24) $$\mu =Y\hat{\xi }$$

where μ [1/h] represents the specific growth rate and *Y* [mol biomass/mol-glucose] is the yield coefficient of biomass on glucose, i.e., the stoichiometry coefficient associated with the biomass in the growth equation (Equation (8)).

In the process of evolution, the rate of entropy production is increased. The rate of entropy production (see Equation (12)) can be increased (i) by increasing the specific glucose uptake rate or (ii) by changing the rate structure of the network such that a higher specific entropy of reaction is obtained. The latter case would require that more weight (usage probability) would be given to an elementary mode that has a higher entropy of reaction. The highest rate of entropy production is obtained when the highest specific glucose uptake rate is reached together with the associated rate structure of the metabolism. In that case the specific glucose uptake rate becomes ${\hat{\xi }}_{max}$ and the network structure is given by

(25) $$p_{j}=\frac{\exp\left(-\frac{{\hat{\xi }}_{max}∆s_{j}}{QR}\right)}{Z}$$

representing the most probable distribution of elementary modes for the case of a fully evolved metabolic network.

But there could be the case where this state of ultimate network structure has been reached but not yet the state of maximum specific glucose uptake rate. In such a case, the specific glucose uptake rate of a cell can be increased, in principle, by increasing in equal proportions all catalysts (enzymes) in a cell. This would increase proportionally the rate of each individual reaction including the specific growth rate, without changing the network structure. But one should expect a limit to this increase, since there will likely be a maximum specific glucose uptake rate that a cell can achieve due to physical transport limitations. For instance, glucose can only diffuse to the surface of a cell at a maximum rate dictated by the diffusion coefficient, or glucose uptake could be limited by a limited number of permeases on the cell surface [33]. It is therefore useful to relate the experimentally measured glucose uptake rate to this maximum possible specific uptake rate

(26) $$\hat{\xi }=b{\hat{\xi }}_{max}$$

where ${\hat{\xi }}_{max}$ [mol glucose/h.g CDW] is the maximum specific glucose uptake rate of a cell attainable by evolution under the given environmental conditions, and b represents the fraction of the maximum specific growth rate that the strain has attained during the ongoing evolution process.

Thus, the usage probability of an elementary mode of a *fully evolved* metabolic network structure in a cell that has not yet attained the maximum specific glucose uptake rate, can be computed explicitly from

(27) $$p_{j}=\frac{\exp\left(-\frac{\hat{\xi }∆s_{j}}{bQR}\right)}{Z}$$

or, in linearized form, from

(28) $$\ln p_{j}=-\frac{\hat{\xi }∆s_{j}}{bQ\;R}+c$$

If we know the maximum possible glucose uptake rate, we can estimate b from the measured actual specific glucose uptake rate using Equation (26). Alternately, if we do not know the maximum possible macroscopic glucose uptake rate, we need to determine both the specific glucose uptake rate at the current point in the evolution process and estimate the fraction b as shown below.

In case the internal rate structure is determined by the type 1 entropy production rate, an analogous expression is obtained in which the reaction entropy is substituted by the affinity of reaction divided by T. However, we will focus in the following on the type 2 entropy production rate as the data suggest that this type determines the usage frequencies of elementary modes, the justification for which we present later in the Discussion.

### 2.2. Comparison of Theory with Experimental Systems

Recently, a radioisotope labeling method combined with mass spectrometry has been developed that allows estimation of multiple intracellular reaction rates comprising a metabolic reaction network [34]. This method has been applied to evaluate the reaction rates of six strains of *E. coli* that were evolved over 300 generations from the same ancestor cell line in growth experiments using glucose as the carbon and energy source [35]. Over this time the strains increased their specific growth rate by 28–38%. Surprisingly, in the set of evolved strains, the network structure is very similar to the original wildtype. This could indicate that the strains are already close to the fully evolved network state. We have used these data to test the presented theory. We first determined the constant b from the experimentally measured glucose uptake rate and Equation (28), and then predicted the intracellular rate structure and compared it with the experimentally measured data.

**Figure 1:**
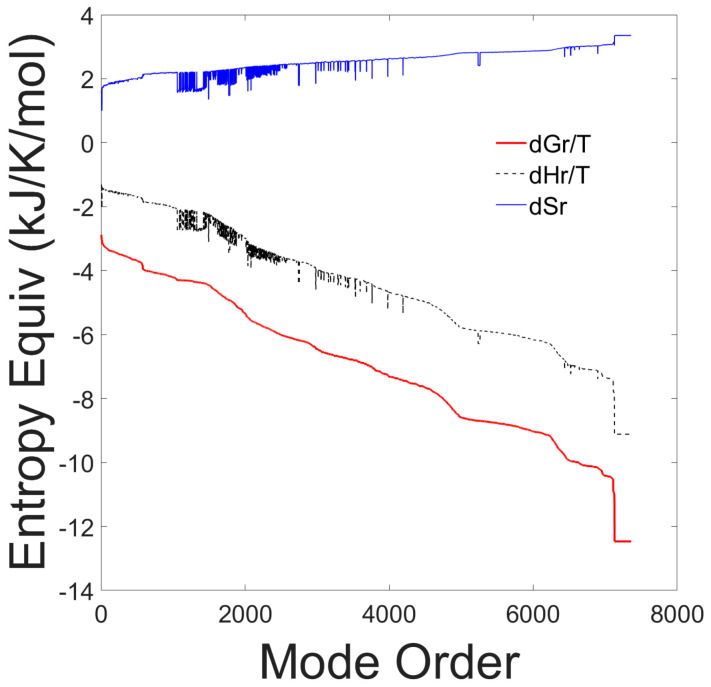
Entropy equivalents from Gibbs free energy, enthalpy and entropy of reaction of all 7363 elementary modes. The thermodynamic properties are plotted as a function of the elementary mode number after sorting the modes according to decreasing Gibbs free energy values.

The thermodynamic reaction properties for each elementary mode, sorted according to decreasing values of Gibbs free energies of reaction, are shown in Figure 1.

The graph shows that the Gibbs free energy of reaction is negative for all elementary modes, indicating that all elementary modes are thermodynamically spontaneous at the experimental conditions. Furthermore, all elementary modes are exothermic. There is a strong correlation between the Gibbs free energy and the enthalpy of reaction and an inverse correlation with the entropy of reaction. All elementary modes have a positive entropy of reaction and a negative Gibbs free energy of reaction indicating that all pathways are feasible and spontaneous. The results of this first test, are presented to justify our assumption that all identified EMs should be included in the probability distribution.

**Figure 2:**
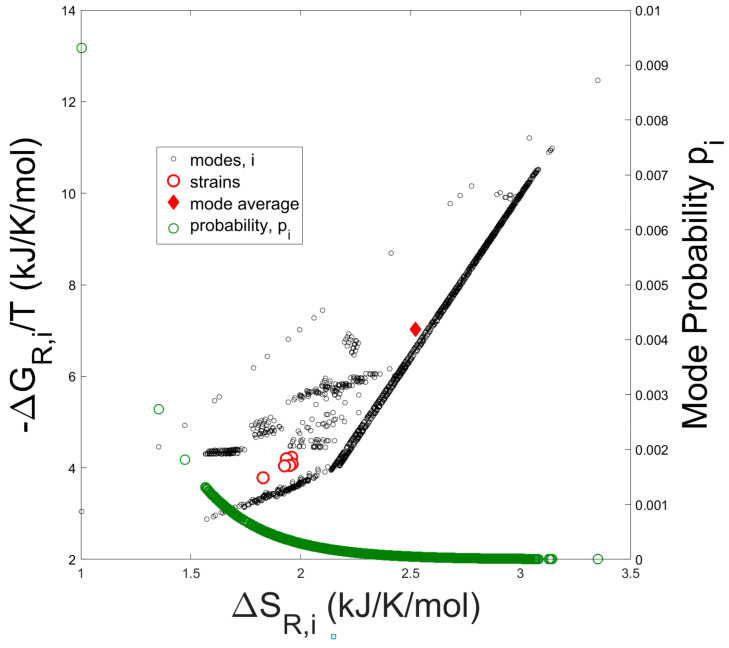
Negative Gibbs free energy of reaction divided by temperature vs. Entropy of reaction for all 7363 elementary modes. The solid diamond represents the arithmetic average over all elementary modes. Open red circles represent the thermodynamic reaction values computed from experimental measurements of externally occuring metabolites (e.g., oxygen, CO_2_, biomass, and acetate). Open green circles represent the usage probabilities of individual elementary modes computed as in Figure 3.

In Figure 2 the entropy contribution from the Gibbs free energy of reaction (−∆G_j_/T) versus the entropies of reaction (∆S_j_) are plotted for all elementary modes.

The solid diamond represents the arithmetic average of −∆G_j_/T and ∆S_j_ taken over all modes, while the open red circles represent the entropy and Gibbs free energy of reaction computed from the experimentally determined overall growth reaction (Equation (8)) of each strain. Thermodynamic values were calculated from the experimental flux data for the externally occurring metabolites of the seven strains tested by Long [35]. Compared to the mode-average entropy and Gibbs free energy of reaction, which assumes a uniform probability distribution over all the modes, the experimentally determined thermodynamic values are clearly biased towards lower values of entropy as expected from a Boltzmann distribution of elementary modes (see Equation (28)). In addition, probability values for each elementary mode are superimposed onto the figure.

**Determination of the maximum glucose uptake rate from experimental data**

The metabolic network contains n Elementary Modes (n = 7363), which were computed using Cell Net Analyzer, based on the reaction network specified by Long et al. The elementary modes are listed in Supplementary File S2. Based on n elementary modes, Equation (28) results in n independent relationships in which the probabilities p_j_ and the constants b and c account for n + 2 unknowns. Thus, to completely specify the system, two additional relationships are needed. One is given by the requirement that the probabilities must sum up to unity, and the second is provided from the experimental data for the stoichiometry coefficients of the overall growth reaction (Equation (8)) that permits computation of the entropy of reaction according to Equation (10). The solution is numerically accessible in MATLAB (R2019a) using the Levenberg–Marquardt least square algorithm for solving non-linear equations.

Note that each EM can be represented by a reaction equation as shown in Equation (8) that is based only on external metabolites. Only the stoichiometry coefficients may be different. Then, the Gibbs free energy and entropy of external metabolites can be evaluated using standard physical chemistry approaches, and, from the difference in products and reactants, the Gibbs free energy and entropy of reaction can be evaluated. These values are then used in the system of equations as described above and numerically solved using MATLAB providing the probabilities of each EM and the parameters of the distribution. Once the parameters of the distribution are known, the probabilities can be directly computed from Equation (27) or (28).

**Figure 3:**
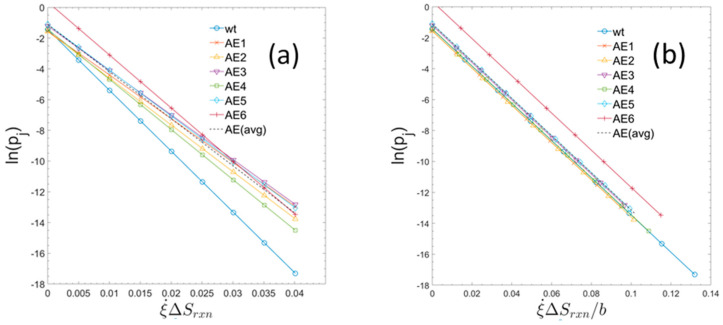
Usage probabilities of elementary modes as a function of entropy production rates of individual elementary modes for the six evolved strains and for the parent wildtype. (**a**) For each strain the usage probabilities of elementary modes and the constants b and c have been computed (see text). The set of data points show the linear relationship defined by Equation (28). (Rather than plotting all 7363 probabilities for each strain only a set of equally spaced datapoints covering the range of entropy production rates is displayed.). The single, dashed line is the computed linear regression of all the displayed datapoints from each strain. (**b**) In the right-hand graph, the entropy production rates are normalized to the maximum glucose uptake rate by dividing by the unique value of b for each strain. Presented in this way, the slope of each graph represents the universal gas constant (or molar Boltzmann constant). AEx = ALE-x (as used in text).

Figure 3 shows the usage probabilities of elementary modes as a function of specific entropy production rates (type 2). When the entropy production values for the strains are normalized, each by their own specific value of b, the trends all collapse to a common form, having the same slope which corresponds to the universal gas constant as defined by Equation (20). The measured macroscopic growth parameters together with the constants b and c for the individual strains are summarized in Table 1.

Using Equations (24) and (26) along with the measured specific growth rate for each strain and the computed constant b, we can make an estimate of the maximum theoretical growth rate (μ_max_) that is possible for *E. coli* under the given environmental conditions. Then, with the maximum specific growth rate, the minimum doubling time can also be calculated (see Table 1).

The average minimum doubling time for all strains is 18 ± 1.4 min. This predicted doubling time points to further evolution capacity as it is shorter than the doubling time of 23 min inferred after evolving *E. coli* on minimal media over 21 years or 50,000 generations [36]. In making this calculation, it is important to remember, we have assumed that all strains are already at (or very near) the optimum network structure. Consequently, this implies that the additional rounds of adaptive evolution conducted by Long et al., served mainly to reduce the effects of any rate limiting states that were restricting the overall flux of glucose, rather than affecting any significant changes in the distribution of the underlying, elementary modes.

**Estimation of measured fluxes and comparison to predicted fluxes**

With the maximum possible glucose uptake rate identified we can now explicitly compute the usage probabilities of elementary modes from Equation (28). Since each elementary mode is assumed to be operating at steady state, without accumulating intermediate metabolites, the metabolic flux across all reaction steps of the EM is conserved. The flux contribution for each elementary mode can then be obtained from Equation (9), and the rate of production of each external metabolite can be computed from

(29) $$r_{i}=\overset{n}{\underset{j=1}{\sum }}\nu_{i,j}{\dot{\xi }}_{j}$$

where r_i_ [moles/h] represent the rate of change in the overall chemical equation and ν_j,i_ the stoichiometry coefficient of the ith reactant of elementary mode j and its corresponding flux ξ_j_. The rates of internal reactions can be obtained from

(30) $$r_{k}=\overset{n}{\underset{j=1}{\sum }}p_{j}{\dot{\xi }}_{k,j}$$

where r_k_ [mol/h] is the flux through the kth reaction in the metabolic reaction network and ξ_k,j_ is the contribution to the kth reaction by elementary mode j.

**Figure 4:**
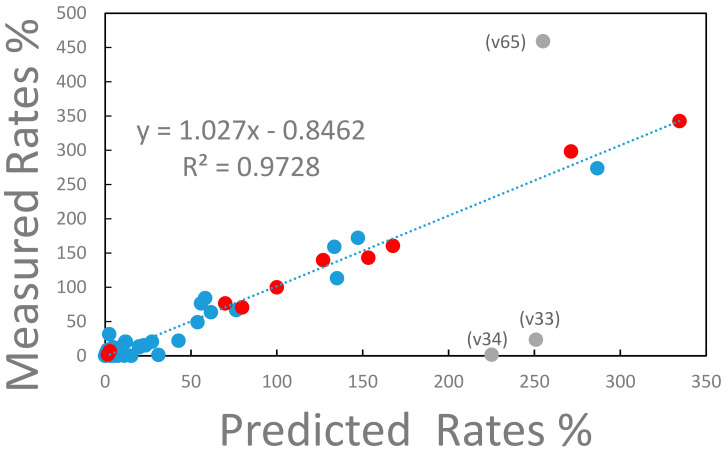
Measured vs. predicted metabolic rates for strain ALE-3. The rates of the metabolic reaction network are expressed relative to the glucose uptake rate, which has a value of 100%. The metabolic reaction network consists of 73 reaction steps of which 11 (marked with red full circles) are transport reactions into and out of the external cell environment. The remaining intracellular reactions are marked with closed blue circles. The three labeled, gray datapoints deviate significantly from the correlation between measured and predicted reaction rates. Label v65 represents the reaction rate of the conversion of intracellular ATP into the external ATP pool (see text for explanation). Label v34 represents the anabolic conversion of oxaloacetate to phosphoenolpyruvate consuming ATP. Label v33 represents the anaplerotic conversion of phosphoenolpyruvate into oxaloacetate. The linear regression represented by the dashed line, excludes the labeled datapoints.

The experimentally determined reaction rates for strain ALE-3, which is representative for all other strains, are compared with the reaction rates predicted from the model in Figure 4.

With the exclusion of three outlying flux values (v33, v34, and v65), the measured and predicted reaction rates are in remarkable agreement as expressed in the R^2^ value of 0.97 of the linear regression of the data (see Figure 4). The three, inconsistent reaction rates represent (i) the conversion of ATP into the external ATP pool (label v65 in Figure 4), (ii) the anabolic conversion of oxaloacetate into phosphoenolpyruvate consuming ATP (label v34 in Figure 4), and (iii) the anaplerotic conversion of phosphoenolpyruvate to oxaloacetate (label v33 in Figure 4). The discrepancy arises because in the measured data set assumes a significant export of ATP and the anaplerotic reaction v34 is essentially zero while the elementary mode-based model predicts a significant reaction activity of v34 that consumes ATP. Thus, in the model, based on elementary modes, a significant turnaround between phosphoenolpyruvate and oxaloacetate consuming ATP is predicted. If this ATP consumption would be assigned to a maintenance reaction (not included in the model) the experimental data would be very well predicted by the model considering that the total consumption of ATP is within 4% when comparing the experimental data with the prediction. At this point it is not clear whether this discrepancy is possibly caused by the extraction of the rate data from the measurements. For instance, a constant ATP production based on a P/O ratio of 2 has been assumed for all strains in the experiment (Long et al., 2017). Or errors could be introduced when a futile cycle exists in the model of elementary modes which lacks a reaction consuming energy for cell maintenance.

## 3. Discussion

Starting from the basic material, energy and entropy balances of a precisely defined system, a continuous stirred tank reactor (CSTR), we provide a theoretical justification for the Maximum Entropy Production principle when the system is in a stationary state that minimizes its Gibbs free energy. We show that in this state the Gibbs free energy of the system is proportional to the negative rate of entropy production, especially for conditions when there is a minimal change in state between the outlet and the inlet conditions, e.g., isothermal operation with low fractional conversion of the incoming stream. In such reacting system the chemical forces drive the system into a time invariant steady state in which the Gibbs free energy is at a minimum. Therefore, the entropy production rate must be at a maximum. The presented derivation is striking due to its simplicity and is a direct extension of the Gibbs free energy concept [7] to a non-equilibrium situation at steady state [8].

The macroscopic relations show that the Gibbs free energy of the system is at a minimum when the rate of entropy production (type 1) is maximized. In contrast, the entropy of the system is at a maximum when the rate of entropy production (type 2), which is based only on reaction entropies, is maximized. Thus, the question arises whether evolution attempts to minimize the Gibbs free energy content of the system or to maximize the entropy content of the system. In order to test this question, we compared flux estimates obtained experimentally to flux estimates calculated using the thermodynamic properties of the discrete reaction trajectories of the metabolic network. The described system is unique in the sense that all discrete reaction states can be computed, and the associated distribution can be solved numerically. In many other cases the distribution of microscopic states can only be inferred based on assumed maximization principles such as the maximum entropy principle (see for instance [37,38]). The obtained frequency distribution functions are either defined by the affinities (negative Gibbs free energies) or by the entropies of reaction of the individual trajectories. It turns out that both approaches provide frequency distributions that can fit the experimental rate data. This is expected since the reaction affinities are strongly correlated with the reaction entropies (see Figure 2). Thus, the reaction entropies as a random variable can be linearly transformed into another random variable, the reaction affinities, resulting in similar frequency distributions. However, there are significant differences between the results of the two models. First, when the three inconsistent reactions are removed, the fit between measured and predicted internal and external reactions is better for the reaction entropy model than for the reaction affinity model (R^2^ = 0.97 vs. R^2^ = 0.94). Furthermore, the slope of the correlation between the two datasets is close to 1 for the reaction entropy model (1.02) in contrast to 0.95 in the case of the reaction affinity model. Second, there is a significant difference in the prediction of the maximum possible specific growth rate. The frequency distribution model that is based on reaction entropies predicts a shortening of the doubling time during the progressing evolution process consistent with the selection process that requires increasingly faster specific growth rates. In contrast, the reaction affinity-based model predicts a doubling time that is longer than the current one. This would require slower specific growth rates when the system evolves which is not consistent with the selection mechanism. Therefore, the former model is expected to apply.

But this does not contradict the MEP principle and the minimization of the Gibbs free energy of the system at steady state. This state is achieved by the variation in reactant concentrations in the system. In contrast, the entropy of the system is maximized through variation in the frequencies that individual elementary modes are used in the metabolism. Evolution appears to favor this state which seems to be a general property as it can be observed not only in the metabolic network of *E. coli* but also in the metabolism of other bacteria such as *Thermoanaerobacterium saccharolyticum* [26]. One should note also that the difference between the two models is generated by the heat effect of the reactions. If there are no heat effects involved in the reacting system, the two approaches become equivalent.

The developed framework provides a possible explanation for the evolution of regulation in the metabolism. One can argue that it is driven by the tendency of the system to operate at the state of the most probable distribution of elementary modes. Therefore, individual enzymes have to be expressed in coordination to support such distribution. Regulation of enzyme synthesis is thus determined by the underlying thermodynamic principles of a reacting network to maximize the entropy production of the system. This has important implications in biotechnology where production systems are created that are expected to be efficient and robust. Furthermore, this opens a much broader outlook on explaining biological behavior and evolution. What happens when environmental conditions change? Does the system evolve towards the new set of conditions? And do these principles also apply to more complex organisms? These are testable propositions which will have to be addressed in future work. Thus, an important goal for future research is to elucidate how complex gene regulatory networks evolve and how their evolution results in phenotypic change and speciation [39].

Recently, a global non-equilibrium thermodynamics for stationary states has been developed and extended to a two component photochemical reaction. The authors point out that one important element missing in non-equilibrium thermodynamics is the variational principle [40]. But this principle is demonstrated in our work for a much more complex biological reaction system represented by the elementary modes of metabolism.

The idea of using the Second Law of thermodynamics to describe evolution is not new. As far back as in the 19th century, physicist Ludwig Boltzmann was contemplating about connections between Darwin’s theory of evolution and statistical thermodynamics [41]. The connection between increasing entropy and decreasing Gibbs free energy, provides a unified understanding of how metabolism and nature works.

## 4. Methods

The creation of adaptive laboratory evolved *E. coli* strains (ALE 1-6), as well as measurements of their growth rate, biomass yield, and external metabolite stoichiometries (glucose, acetate, and oxygen) were reported by Sandberg [42]. Measurements of internal fluxes, using ^13^C radioisotope labeling, and creation of the reaction network model used for metabolic flux analysis were completed by Long et al. [35].

In the present study, the original reaction network that was specified by Long et al. has been adapted with the following modifications: (i) two new equations are added, v72, for the export of water (h2o → h2o.Ext) and v73, for the import of phosphate (po4.Ext → po4); (ii) Where appropriate h2o, po4, NAD, NADP, and CoA are added to the original equations in order to satisfy elemental balances on C, H, N, O, P, and S, and (iii) Equation v24 (1 akg + 1 coa + 1 nad => 1 succoa + 1 co2 + 1 nadh) has been changed from reversible to irreversible (which is in accord with the latest scientific information). In addition, a proton balance reaction has been added to the model (v74). These modifications, particularly (i) and (ii) are necessary to obtain correct estimates for the thermodynamic reaction properties of each individual elementary mode and of the overall growth reaction. A list of the metabolic reactions used in the model is given in the Supplementary Materials S2. Elementary modes of the network (7363 total) were computed using CellNet Analyzer [43], available for download at www2.mpi-magdeburg.mpg.de/projects/cna/cna.html). A table of all the elementary modes is included in the Supplementary Materials S2.

To make an accurate calculation of the thermodynamic properties of each elementary mode, it is necessary to make an estimate of the concentration of external metabolites present during cultivation. To obtain these values, we constructed a mathematical model of the cell evolution experiment based on approximation of each culture as a continuously stirred tank reactor (CSTR), operating at steady-state conditions. Parameter values for the CSTR simulation were taken from the growth rate and stoichiometric yield data collected by Sandberg et al. To summarize; strains were cultivated in flasks with 15 mL of M9 media, supplemented with 2 g/L glucose, kept at 37 °C and mixed well to provide full aeration. To maintain the cells in a continual state of exponential growth and avoid glucose depletion, cultures were grown in a semicontinuous fashion with repeated transfers into fresh media. Prior to transfer, cells were cultivated to an optical density of ~1 (OD_600_) before being diluted into fresh media (100 μL into 15 mL). Using an automated transfer system, cell cultivation was continued for ~1000 generations before the evolution was concluded [42]. A detailed description of the CSTR model is given in the Supplementary File S3. The steady-state concentrations of the metabolites together with the corresponding values of the thermodynamic properties of formation are summarized in Supplemental File S4 for a medium conversion condition that has been applied in the presented analysis.

For the most accurate estimate of thermodynamic properties of biological molecules, Alberty has pointed out that calculations should consider not only factors such as concentration and temperature, but also pH, ionic strength, concentrations of metal cofactors (e.g., Mg), and the potential for certain compounds to exist simultaneously in multiple, ionic charge states (e.g., aqueous phosphoric acid occurring as H_3_PO_4_, H_2_PO_4_^−1^, HPO_4_^−2^, and PO_4_^−3^, together referred to as a “*pseudoisomer*” group) [44]. Furthermore, Alberty has proposed that the thermodynamics properties of pseudoisomer groups can be modeled using a single, weighted average, with the weighting factor for a specific pseudoisomer *i* being calculated as *p_i_ = exp ((μ_iso_ − μ_i_)/RT).* Here, *μ_i_* is the chemical potential of the i-th pseudoisomer, and *μ_iso_* is the composite average of the pseudoisomer group. (Interestingly, the probability function derived by Alberty to explain the distribution of pseudoisomer species is very similar in form to the one we have derived in Equations (16) and (17) for describing the probability distribution of elementary mode usage.) Values for enthalpy and free energy of formation of chemical species at the standard reference state were obtained primarily from Atkins [45] and Alberty [44]. Other sources include: Rard and Wolery [46] for phosphate and Roels [47] for biomass. The molecular formula for *E. coli* biomass was taken as CH_1.6_N_0.26_O_0.38_P_0.023_S_0.006_ [48]. While we did include pH and ionic strength as part of our thermodynamic model, we did not include the effects of Mg^2+^ ions.

In the present paper, we extend the computation of pseudoisomer average thermodynamic properties to include not only the *aqueous* pseudoisomers but also the components present in *gaseous* form (e.g., H_2_O_g_, O_2,g_, and CO_2,g_). Because the adaptive evolution experiments conducted by Sandberg et al. were performed in well-mixed flasks, we have assumed that both oxygen and carbon dioxide participate primarily as gas phase reactants. However, for water, which has a lower volatility, the CSTR model predicts that only ~4 mol% of this species is present in the gas phase. Thus, the thermodynamic values for water are computed as a molar average of both gas and liquid properties.

Recently, the method of pseudoisomers has been taken up by Noor et al.*,* who have curated a collection of thermodynamics properties for different ionic species and has made them available via an online database and thermodynamic calculator, the *Equilibrator* [49]. While in the present study we use our own routine for calculating non-reference-state thermodynamic properties and pseudoisomer averages, comparison of our values with those available from the *Equilibrator* gives largely identical results.

Another useful result that comes from the pseudoisomer calculations is an estimate for the average *charge state* of each ionizing species, which is a function of both the pH and the ionic strength of the media. Ionizing species that occur in the elementary mode equations include ammonia, acetate, phosphate, sulfate, carbonate, and water. By performing a charge balance on each mode, the number of protons required for charge neutrality can be calculated and then incorporated into the overall stoichiometric equation for each mode. Because protons are included in the net stoichiometric equation, they must also be incorporate for computation of each mode’s reaction thermodynamic values. For this calculation, we assumed that pH remains constant at a value of 7.0 throughout cultivation, which is reasonable because of pH buffering by the medium and, because media depletion is minimized by keeping cultures in a low-density state. However, for a neutral pH, the formation enthalpy and free energy values of protons are zero; thus, at pH 7 the production of protons by an elementary mode does not affect its reaction thermodynamics values. Nevertheless, the number of protons produced by each mode is still a useful value, because it gives an additional flux for evaluating agreement between experiment and the model. For biological systems, consideration of pH effects can be especially important because several biologically relevant compounds have acidic isoforms, which when present at the standard, thermodynamic, reference concentration of 1 M, result in pH levels very far from neutral, e.g., ammonia (pH = 11.4), carbonate (pH = 3.18), acetate (pH = 2.38), lactate (pH = 1.93), phosphate (pH = 1), and sulfate (pH = 0).

Experimental data from Long et al. [35] and thermodynamic reference data were stored in Microsoft Excel. All other computation, data analysis, and figure generation were made using Matlab R2019a. A pseudocode of the important computational steps is provided in the Supplemental File S5.

## Figures and Tables

**Table 1:** Measured growth parameters [35] and computed constants b and c.

	μ	q_s_	Y_b/g_	b	c	μ_max_	q_s, max_	τ_min_
	1/h	mmol/h.gCDW	g/g	―	―	1/h	mmol/ h.gCDW	min
WT	0.670 ± 0.002	8.46 ± 0.42	0.444	0.30410	−1.44	2.23	−27.83	19
ALE-1	0.886 ± 0.030	11.57 ± 0.52	0.425	0.42324	−1.55	2.09	−27.35	20
ALE-2	0.869 ± 0.015	10.79 ± 0.40	0.447	0.39574	−1.57	2.20	−27.27	19
ALE-3	0.891 ± 0.024	11.97 ± 0.72	0.414	0.41514	−1.20	2.15	−28.82	19
ALE-4	0.819 ± 0.013	10.29 ± 0.27	0.442	0.36869	−1.42	2.22	−27.91	19
ALE-5	0.934 ± 0.011	11.82 ± 0.57	0.439	0.40408	−1.11	2.31	−29.26	18
ALE-6	0.936 ± 0.021	12.46 ± 0.37	0.417	0.34908	0.35	2.68	−35.69	16

μ measured specific growth rate, [1/h]. q_s_ measured glucose uptake rate, [mmol/h.gCDW]. Y_b/g_ measured yield of biomass on glucose [g/g]. μ_max_ maximum specific growth rate, predicted [1/h] (μ/b). q_s, max_ maximum glucose uptake rate, predicted, (q_s_/b) [mmol/h.gCDW]. τ_min_ minimum doubling time, predicted ($\ln\left(2\right)/\mu_{max}$) [min].

## Data Availability

The data have been submitted in the Supplemental Files.

## Supplementary Materials

The following supporting information can be downloaded at: https://www.mdpi.com/article/10.3390/metabo15030200/s1.
