# Supplementary material for: Predicting the Rate Structure of an Evolved Metabolic Network
Source: Metabolites. 2025 Mar 13;15(3):200. doi: 10.3390/metabo15030200 (PMC11944149; doi:10.3390/metabo15030200)
Supplement: Supplementary file 1 [file metabolites-15-00200-s001.zip › S1.pdf]

Non-equilibrium Thermodynamics of open systems (detailed derivation)

Consider an open system such as a continuous stirred tank reactor (CSTR) with constant volume V and operating at constant temperature T and pressure p. The composition of the entering stream is a parameter of the system, while the composition of the outflowing stream is a dependent variable subject to the thermodynamic state of the system. The values (or) parameters associated with the incoming stream are marked with the suffix 'in'. The outgoing stream is of the same composition as the internal system since it is well mixed and homogeneous. Outgoing streams are not ascribed a suffix. Heat can be exchanged through the surface of the system. To simplify the balances we assume that the entering stream has the same temperature as the system and the CSTR is operated isothermally. In this system cell growth occurs. The incoming stream contains all the required nutrients and the outgoing stream contains unreacted nutrients, cells and secreted products.

It is useful to review the basic material, energy and entropy balances for such system. This introduces and defines in detail the variables and parameters of the presented framework and provides a comprehensive summary of the approach taken.

Material Balance. The material balance for such open system can be written for each component as follows

$$\frac{dn_i}{dt} = \left(\dot{n}_{i,in} - \dot{n}_i\right) + V\nu_i\dot{\xi} \qquad (1)$$

where $\dot{n}_i$ are the molar flow rates of each component i, $\dot{\xi}$ [moles/L.hr] is the extent of reaction and ν_i is the stoichiometry coefficient of each component as it appears in the growth equation, and V [L] is the reactor volume. Microbial cells in the reactor, lumped together as "biomass", are treated similarly to other product molecules excreted by the cell. Using empirically determined values of

molecular composition, enthalpy of formation, and entropy of formation, a generic biomass molecule is defined, which is easily incorporated into standard thermodynamics calculations While biological cells do rely on a multitude of internal metabolites, for the purpose of thermodynamic calculations, it is necessary to track only those that cross the cell membrane. Only nutrients that are taken up by cells and products that are secreted by the cells have to be considered in the balance to completely describe the system. This makes for a convenient simplification in the macroscopic description, since much of the complexity of the metabolism disappears as it is contained within the cells.

Energy Balance. To obtain an expression for the enthalpy of reaction we multiply Equ. (1) with the molar component enthalpies, h_i and obtain for steady state conditions the expression

$$0 = h_i(\dot{n}_{i,in} - \dot{n}_i) + V\dot{\xi}\nu_i h_i \tag{2}$$

If we sum up the component balance equations over all species, i, we obtain

$$0 = \sum_i h_i(\dot{n}_{i,in} - \dot{n}_i) + V\dot{\xi}\sum_i \nu_i h_i \tag{3}$$

We define the enthalpy of reaction ΔH_R

$$\Delta H_R \equiv \sum_i \nu_i h_i \tag{4}$$

and substitute in (3) to obtain after rearrangement

$$\sum_i h_i(\dot{n}_{i,in} - \dot{n}_i) = -V\dot{\xi}\Delta H_R \tag{5}$$

This relationship defines the rate of enthalpy production as the difference in enthalpy content of the inlet and outlet streams.

The energy balance under the assumed simplifying conditions can be written as

$$\frac{dH}{dt} = \sum_i h_i\left(\dot{n}_{i,in} - \dot{n}_i\right) - \dot{Q} \tag{6}$$

where H is the enthalpy content of the system, h_i are the molar enthalpies of the individual components, $\dot{n}_{i,in}$ and $\dot{n}_i$ are the molar flow rates in and out of the system and $\dot{Q}$ is the rate of heat transfer to the environment. Note, because the system outlet is constrained to the same temperature as the system inlet, $h_{i,in} = h_{i.}$ If we substitute (5) in (6) we obtain at steady state

$$\dot{Q} = -V\dot{\xi}\Delta H_R \tag{7}$$

Entropy Balance. To obtain an expression for the entropy of reaction we multiply the steady state material balance (1) with the molar component entropy s_i

$$0 = s_i(\dot{n}_{i,in} - \dot{n}_i) + V\dot{\xi}\nu_i s_i \tag{8}$$

If we sum up all component equations we obtain

$$0 = \sum_i s_i(\dot{n}_{i,in} - \dot{n}_i) + V\dot{\xi}\sum_i \nu_i s_i \tag{9}$$

We define the entropy of reaction ΔS_R

$$\Delta S_R \equiv \sum_i \nu_i s_i \tag{10}$$

and substitute in (9) to obtain

$$\sum_i s_i(\dot{n}_i - \dot{n}_{i,in}) = V\dot{\xi}\Delta S_R \tag{11}$$

We formulate a balance for entropy in a similar way as previously for the other extensive quantities, except we have to consider that entropy of a component depends also on its concentration. To account for this we write

$$\frac{dS}{dt} = \sum_i (s_{i,in}\dot{n}_{i,in} - s_i\dot{n}_i) - \frac{\dot{Q}}{T} + \dot{S}_{gen} \tag{12}$$

Here $s_{i,in}$ (s_i) are the molar entropies of the individual components at the corresponding concentrations transported in (out) of the system; $\dot{Q}$ is the rate of heat transfer through the walls, and $\dot{S}_{gen}$ is the rate of internal entropy generation of the system due to the irreversibility of the process. The first two terms on the right hand side represent the net entropy transported to the surroundings due to material transport and due to heat transfer, respectively. From this expression one can see that in a steady state situation with zero entropy accumulation, the internal entropy production term must be balanced by the transport of entropy to the surroundings. Due to the second law, the internal entropy production term must always be larger or equal to zero.

There are two significant irreversibilities contributing to the internal entropy generation, the entropy of mixing and the entropy of reaction

$$\dot{S}_{gen} = \dot{S}_{mix} + \dot{S}_{rxn} \quad (13)$$

The entropy of mixing is generated by the instantaneous dilution of the incoming stream to the concentrations inside of the ideal CSTR

$$\dot{S}_{mix} = \sum_i \dot{n}_{i,in}(s_i - s_{i,in}) \quad (14)$$

The rate of entropy production due to the irreversibility of the reaction is related to the affinity of reaction A (De Donder & Van Rysselberghe, 1937):

$$V\dot{\sigma}_{rxn} = \dot{S}_{rxn} = V\frac{A}{T}\dot{\xi} \quad (15)$$

Where $\dot{\sigma}_{rxn}$ [J/K.l.hr] is the rate of entropy production per unit volume, and A [J/mole] is the affinity of reaction defined as the negative of the Gibbs Free Energy of reaction

$$A \equiv -\Delta G = -\Delta H + T\Delta S \quad (16)$$

The Gibbs Free Energy of reaction is related to the enthalpy and entropy of reaction by the Gibbs relationship (Equ.16). Combining Equs. 13-16, the rate of entropy production due to the irreversibility of the reaction system then becomes

$$\dot{S}_{gen} = \sum_i \dot{n}_{i,in}(s_i - s_{i,in}) + V\dot{\xi}\left(\frac{-\Delta H}{T} + \Delta S\right) \quad (17)$$

Substituting in the entropy balance (12) we obtain

$$\frac{dS}{dt} = \sum_i s_i(\dot{n}_{i,in} - \dot{n}_i) - \frac{\dot{Q}}{T} - V\frac{\Delta H}{T}\dot{\xi} + V\Delta S\dot{\xi} \quad (18)$$

And substituting for the rate of heat transfer (Equ.7) at steady state results in

$$0 = \sum_i s_i(\dot{n}_{i,in} - \dot{n}_i) + V\Delta S\dot{\xi} \quad (19)$$

We can substitute the molar flow rates $\dot{n}_i$ with the product of volumetric flow rates F [L/hr] and concentrations c_i [mole/L] and introduce the space time $\tau =$ V/F [hr] to yield Equ.(20). Recalling that entropy is an *extensive* property, we see that the expression provides a statement of the entropy content of the system at steady state

$$S_{sys} = \sum_i s_i\, c_i = \sum_i s_i c_{i,in} + \tau\dot{\xi}\Delta S_R \quad (20)$$

This expression shows that the system entropy is only a function of the entering state (due to the mixing effects) evaluated at the system conditions and the rate of entropy production in the system

contributed by the reaction entropy. Thus, the system entropy is expected to increase as the rate of entropy production by reaction increases. Furthermore, when dilution of the incoming stream is negligible, as is the case for low cell-density cultures, the system entropy approaches a maximum. Using the Gibbs relation, we can convert this equation to obtain an expression for the Gibbs free energy of the system

$$G_{sys} = H_{sys} - \sum_i h_i c_{i,in} - \tau\dot{\xi}\Delta H_R + \sum_i g_i c_{i,in} - T\tau\dot{\xi}\Delta G_R \tag{21}$$

The first three terms on the right-hand side vanish due to Equ. (5) resulting in

$$G_{sys} = \sum_i g_i c_{i,in} + \tau\dot{\xi}\Delta G_R \tag{22}$$

Using Equ (15) and (16) one can also write

$$G_{sys} = \sum_i g_i c_{i,in} - \tau T\dot{\sigma}_{rxn} \tag{23}$$

The Gibbs free energy of the system at steady state is inversely proportional to the rate of entropy production $\dot{\sigma}_{rxn}$. Thus, a reacting open system adjusts the component concentrations such that the Gibbs free energy at steady state is at a minimum due to the tendency to equilibrate chemical potentials. This is accomplished when the rate of entropy production is maximized corroborating the Maximum Entropy Production principle (Martyushev & Seleznev, 2006) (Dewar, 2009). But the corresponding system entropy (Equ. 20) depends only on the rate of entropy formation due to the entropy of reaction. From the contributions to the internal entropy generation expression (see Equ 17) only the reaction entropy affects the entropy of the system. The enthalpic component of the internal entropy generation is exported into the surroundings and does not contribute to the entropy content of the system. The obtained relationships describe the macroscopic behavior of the system and are generally valid for any reacting, non-equilibrium system at steady state.
