# Supplementary material for: Predicting the Rate Structure of an Evolved Metabolic Network
Source: Metabolites. 2025 Mar 13;15(3):200. doi: 10.3390/metabo15030200 (PMC11944149; doi:10.3390/metabo15030200)
Supplement: Supplementary file 1 [file metabolites-15-00200-s001.zip › S3.pdf]

Description of CSTR Model

The procedure used by Sandberg *et al.* for creating the adaptively evolved strains (ALE 1-6) was a semicontinuous cell cultivation. In this procedure, a growing culture is periodically diluted into fresh media to maintain the cells in a state of continuous exponential growth. This technique is often used as an approximation for continuous cultivation experiments, which are more tedious to operate; however, for purposes of mathematical simplicity, we chose to model the evolution experiment as a continuously stirred tank reactor (CSTR).

Solution of the CSTR equations (Eqs. 4) produces a time-invariant steady state that depends on the values of several experiment parameters, including, initial glucose concentration in the media, specific growth rate and stoichiometric yield coefficients for each strain, as well as the initial number of cells (*i.e.* catalyst) seeded into the reactor at the time of startup. If a small number of cells are present in the reactor, the fractional conversion of glucose is low. If the number of cells is large, then the fractional conversion of glucose is high. Although periodic media replacement allows for continual cell growth, the concentration of cells within the reactor (and hence the fractional conversion) is not constant in time, and instead, changes from low to high over each batch cycle. Because of this fluctuation, there is a question as to which fractional conversion (and the resulting metabolite concentration) most accurately define the environmental conditions that influence the cell's evolution.

In the present analysis we chose to model the evolutionary process at conditions near the end of each batch cycle, when fractional conversion is highest. This choice reflects the environment when selective pressure on growth is highest (due to low substrate availability and high metabolite concentrations in the media.)

To determine the fractional conversion and metabolite concentrations at the end of a batch cycle, we model cell growth between media replacements, using a simple, batch-growth model,

	$C_{bio,a} = C_{bio,a}^{inoc} \exp\left(\mu\, t_{exc}\right)$	Eq.1

where $C_{bio,a}^{inoc}$ is the starting cell density, μ is the specific growth rate, t_{exc} is the time between media exchanges, and $C_{bio,a}$ is the cell density just prior to dilution. In the Sandberg paper, data is provided for 1.) The final cell density just before media replacement (~1 OD_{600}), 2.) the specific growth rate and yield coefficients for each strain (various), and 3.) the dilution factor used at the time of media refreshment (100 uL into 15 mL). First, using values from 1 and 3, it is possible to determine the initial cell density after dilution. Then, with Eq.1 we can calculate the time between media refreshments.

As a second relation, we can also express the value of $C_{bio,a}$ in terms of the yield of biomass on glucose $\bar{y}_{bio/glu}$ and the fractional conversion of glucose X_{glu} at the end of the batch

	$C_{bio,a} = C_{glu,a,o} X_{glu} \bar{y}_{bio/glu} + C_{bio,a}^{inoc}$	Eq.2

Combining these two equations and solving for X_{glu} gives

	$X_{glu} = \dfrac{C_{bio,a}^{inoc}}{C_{glu,a,o}\bar{y}_{\frac{bio}{glu}}}\left(\exp(\mu\, t_{exc}) - 1\right)$	Eq.3

Thus, with this formula, a unique value of X_{glu} is determined for each strain. Once we have determined the final fractional conversion using a the batch growth equations, the values of X_{glu}, μ, and the experimentally measured yield vector $\bar{y}_{j/glu}$ are then used to specify a unique CSTR state for each evolved strain.

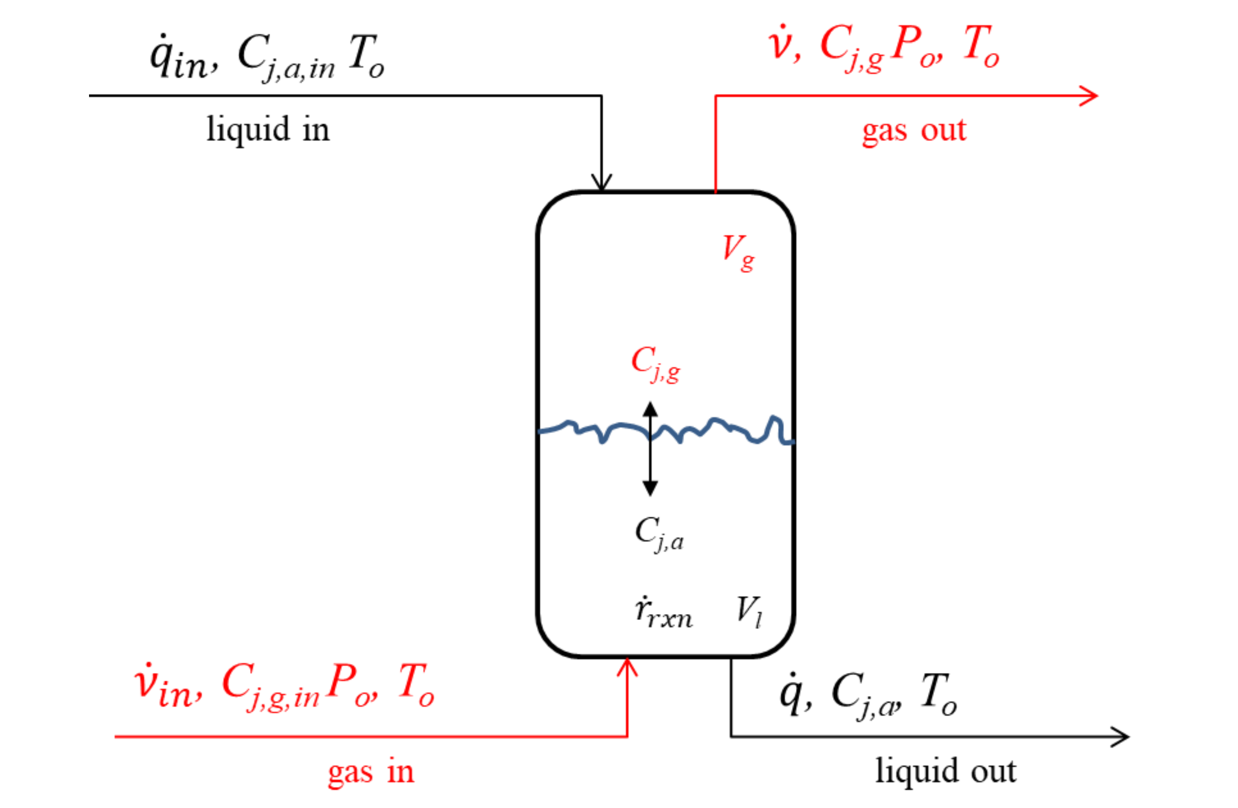

Figure 1. Process Flow Diagram for Combined Liquid and Gas Feed Streams

A hypothetical CSTR is presented in **Figure 1**. The liquid volume inside the reactor is denoted V_l and the gaseous volume is V_g. The liquid flow rates in and out are $\dot{q}_o$ and $\dot{q}$, respectively, while the gas flow rates are $\dot{v}_o$ and $\dot{v}$. Partitioning of volatile species is handled by the use of two concentration variables, one describing the aqueous form of the species ($C_{j,a}$) (mol/L) and the other describing the gaseous form of the species ($C_{j,g}$) (mol/L). For the ψ non-volatile species, j = $\{1,2,\ldots\psi\}$, a total of ψ differential equations are needed. For the ϕ volatile species, j = $\{\psi+1, \psi+2\ldots \psi+\phi\}$, a set of 2ϕ differential equations are needed, one for each phase. Furthermore, 2ϕ

constitutive relations are also needed. The first is for the rate of mass transfer between the two phases , which is modeled with the simple expression $\dot{r}_{xfr} = k_j\left(C^*_{j,a} - C_{j,a}\right)$ where k_j is a lumped mass transfer coefficient (1/h), and $C^*_{j,a}$ is the corresponding concentration aqueous concentration that would be found in equilibrium with 1 mol/L of gaseous substrate, according to Henry's law relation. The second constitutive equation is the Henry's law relation for each volatile species. Lastly, two additional equations are needed to solve for the inlet liquid flow $\dot{q}_o$ and the outlet gas flow $\dot{v}$. For one of these we perform a total mole balance on the entire CSTR, and for the second one, we perform a total carbon balance on the entire CSTR. The model was constructed in MATLAB and integrated using the ode15s solver for simultaneous, non-linear, ODE systems.

(Eqs.4)

$$V_l \frac{dC_{bio,a}}{dt} = V_l\, \mu\, C_{bio,a} - \dot{q}\, C_{bio,a}$$

$$V_l \frac{dC_{j,a}}{dt} = \dot{r}_{glu} V_l \bar{y}_{\frac{j}{glu}} + \dot{q}_{in} C_{j,a,in} - \dot{q} C_{j,a} + \dot{r}_{xfr,j} V_l$$

$$V_g \frac{dC_{j,g}}{dt} = \dot{v}_{in} C_{j,g,in} - \dot{v} C_{j,g} - \dot{r}_{xfr,j} V_l$$

$$\dot{r}_{xfr,j} = k_j\left(C^*_{j,a} - C_{j,a}\right)$$

$$\dot{r}_{glu} = \frac{C_{glu,a,in} X_{glu}}{\tau_l}$$

$$\tau_l = \frac{V_l}{\dot{q}}$$

$$\left(\frac{C^*_{j,a}}{C_{j,g}}\right) = H^{\circ}_j$$

$$\dot{v}_{in} \sum_{j=1+\psi}^{\psi+\phi} C_{j,g,in} + \dot{q}_{in} \sum_{j=1}^{\psi+\phi} C_{j,a,in} + V_l \sum_{j=1}^{\psi+\phi} \dot{r}_{glu} \bar{y}_{\frac{j}{glu}} = \dot{v} \sum_{j=1+\psi}^{\psi+\phi} C_{j,g} + \dot{q} \sum_{j=1}^{\psi+\phi} C_{j,a}$$

$$\dot{v}_o C_{co2,g,in} + \dot{q}_o\left(C_{co2,a,in} + 6C_{glu,a,in}\right) + \\ = \dot{v}\, C_{co2,g} + \dot{q}\left(C_{co2,a} + 6C_{glu,a} + 2C_{ace,a} + C_{bio,a}\right)$$

Model Variables		
	Known (or assumed) Parameters	
$\bar{y}_{\frac{j}{glu}}$	Stoichiometric yield of specie j on glucose.	mol-j/mol-glu
V_l	Volume of reactor liquid phase	L
V_g	Volume of reactor gas phase	L
$C_{j,a,in}$	Concentration of aqueous species j in the inlet liquid	mol/L
$C_{j,g,in}$	Concentration of gaseous species j in the inlet gas	mol/L
T_o	System Temperature. Constant from inlet to outlet	K
P_o	System Pressure. Constant from inlet to outlet	atm
$\dot{v}_{in}$	Inlet gas flow rate	L/h
H_j°	Henry's Law Constant for species j	mol liq/ mol gas
k_j	Overall mass transfer coefficient for species j	1/h
μ	Specific cell growth rate	1/h
$C_{bio,a}^{inoc}$	Concentration of biomass at inoculation	mol/L
	Calculated Variables	
$C_{j,a}$	Concentration of aqueous species in the outlet liquid stream	mol/L
$C_{j,g}$	Concentration of gaseous species in the outlet gas stream	mol/L
$\dot{q}$	Outlet liquid flow rate	L/h
$\dot{q}_{in}$	Inlet liquid flow rate	L/h
$\dot{v}$	Outlet gas flow rate	L/h
t_{exc}	Time period between media exchange.	h
X_{glu}	Fractional conversion of glucose	dimensionless
$C_{j,a}^{*}$	Equilibrium concentration of an aqueous species at *T* and a given partial pressure p_j	mol/L
τ_l	Liquid phase, mean residence time	h
$\dot{r}_{glu}$	Rate of glucose uptake	mol/L/h
$\dot{r}_{xfr,j}$	Rate of liquid/vapor mass transfer	mol/L/h
