# Supplementary material for: Predicting the Rate Structure of an Evolved Metabolic Network
Source: Metabolites. 2025 Mar 13;15(3):200. doi: 10.3390/metabo15030200 (PMC11944149; doi:10.3390/metabo15030200)
Supplement: Supplementary file 1 [file metabolites-15-00200-s001.zip › S5.pdf]

Pseudocode for Calculation of Boltzmann Distribution Parameters from Evolved Strain Data

1. **Import Elementary Modes** Read in list of elementary modes computed from MetaTool using the reaction network specified by Long et al. (2017).

2. **Import Experimental Metabolism Data for Each Strain** including, specific growth rate, overall stoichiometric equation (*i.e.* substrate to product yield coefficients $\overline{\boldsymbol{y}}_{\boldsymbol{p/s}}$), glucose uptake rate ($\dot{\boldsymbol{q}}_{\boldsymbol{glu}}$), and C^{13} flux labeling data for each individual strain.

3. **Specify Process State Variables** Temperature, Pressure, pH, and ionic strength to match the growth conditions used in the experiment.

4. **Import Thermodynamic Quantities (at *Standard* Reference State)** Read in standard reference state, thermodynamic properties of formation (ΔG_f° ΔH_f°, and Henry's law correlations)) for all chemical species. These include aqueous species, gas species, and any associated ions (i.e. CO_3^{-2}). Standard reference conditions are specified as 1 atm, 298 K, and a solute concentration of 1M. Sources for these data include *Physical Chemistry* (Atkins, P*),* the *Equilibrator* (Noor et al., 2012), *Thermodynamics of Biochemical Reactions* (Alberty, R.A.), NIST website, and other research papers in the literature. Data, for ions especially, are obtained from the Alberty book and the *Equilibrator*.

5. **Compute Pseudoisomer Thermodynamic Formation Properties ($\Delta \boldsymbol{G}^*_{\boldsymbol{f,iso}}$) (for *Two-phase Biological* Reference State)** Calculate the composite thermodynamic values for each *equilibrium pseudoisomer* group ($\Delta G^*_{f,iso}$ $\Delta H^*_{f,iso}$, $\Delta S^*_{f,iso}$). These values include contributions from both aqueous pseudoisomer species as well as a (single) gaseous pseudoisomer that is present for some species. In order to calculate the composite liquid-gas thermodynamic functions, it is also necessary to specify the relative volumes of the two phases, $\nu = V_g/V_l$ Note: because the specified reference state of the pseudoisomer groups is one of vapor-liquid equilibrium, the value of $\Delta G^*_{f,iso}$ does not depend on ν; *however,* the values for $\Delta H^*_{f,iso}$ and $\Delta S^*_{f,iso}$ *do* depend on ν. For the reference state we have arbitrarily chosen a value of $\nu = 1$. The other conditions for the *biological* reference state, T,P, pH, and Ionic strength, as chosen to match those of the process, except concentration, which is kept at 1 M and corrected to process conditions at a later step.

6. **Compute Thermodynamic Reaction Functions ($\Delta \boldsymbol{G}^*_{\boldsymbol{R}}$) (for *Two-phase Biological* Reference State)** Calculate the thermodynamic reaction functions at the specified *biological, pseudoisomer, reference state* (ΔG^*_R ΔH^*_R, ΔS^*_R) for each elementary mode (*e.g.* $\Delta G^*_R = \sum_i^{prod} \upsilon_i \Delta G^*_{f,iso,i} - \sum_j^{react} \upsilon_j \Delta G^*_{f,iso,j}$). Note: before computing the thermodynamic functions, the stoichiometry of each mode is adjusted to include three additional effects: 1.) the consumption of additional water needed to form carbonic acid for any mode producing CO_2, 2.) the production / consumption of protons by each mode, and 3.) the addition of OH^- ions (reactant) and H_2O (product) for neutralizing the protons. The calculation of proton production and associated neutralization stoichiometry are needed to satisfy the assumption that pH inside the CSTR remains constant. Additionally, compute thermodynamic reaction functions for each *strain*, instead using the overall stoichiometric equation that was determined from experiment.

7. **Specify Additional CSTR Parameters** including: working volume **V_l** and headspace **V_g,** liquid flow rate $\dot{q}_{liq}$, gas flow rate $\dot{q}_{gas}$, , overall gas mass transfer coefficient $k_l a$, media feed composition $C_{i,liq}^{in}$, gas feed composition $C_{i,gas}^{in}$, cell density at inoculation $C_{bio,liq}^{o}$, cell specific growth rate **μ** (strain specific), yield coefficients for products and substrates $\bar{y}_{p/s}$ (strain specific), and time until media exchange t_{exc}. Combining values of $C_{bio,liq}^{o}$, **μ**, $\bar{y}_{p/s}$, and t_{exc} for each strain, an estimate of the fractional conversion can be made.

8. **Determine CSTR Concentrations** Using the parameters specified above, calculate the *total* concentration of each chemical specie inside the reactor in both the *liquid* and the *gas* phases. Here, *total* means the sum of all the individual pseudoisomers in *one* phase. This calculation is made by solving a dynamic mass balance around the growing culture, which is assumed to operate as an idealized CSTR, including inlet / outlet streams of both media and gas. Separate balances are performed for each specie and for both the gas and the liquid phases, assuming a specified mass transfer coefficient (*kla*) that governs the exchange of gas between them. **See supplementary file S3 for explanation of CSTR model.**

9. **Compute Reaction Functions at *Working* Process Concentrations** Once the steady-state concentrations inside the CSTR are determined, two correction factors are added to the reference thermodynamic function ΔG_R^*. The first is the quantity $RTln(Q^{conc})$ where $Q^{conc} = \prod_i C_i{}^{\nu_i}$ is the usual *reaction quotient.* The second correction is $RTln(Q^{neqss})$, which is used to account for the case when thermodynamic equilibrium between the gas (p_{iso}) and liquid ($C_{aq,iso}$) phases cannot be assumed, for example when the rate of mass-transfer between the two phases is slow. Here, the *neqss quotient (non-equilibrium steady-state*) is defined as $Q^{neqss} = \prod_i \left(K_{H,i}^{iso,*} \frac{p_{iso,i}}{C_{aq,iso,i}} \right)^{\phi_i}$, where $\phi_i = \frac{\nu}{\nu + \frac{C_{aq,iso,i}}{p_{iso,i}} RT}$. From these last two equations, it can be seen that when the gas and liquid phases *are* at equilibrium (*i.e.* $\frac{p_{iso,i}}{C_{aq,iso,i}} = \frac{1}{K_{H,i}^{iso,*}}$ for all species) it must be true that $RTln(Q^{neqss}) = 0$. Thus, if the CSTR is at steady state, but the gas and liquid phases are not in equilibrium, the value of ΔG_R becomes a function of the parameter ν = V_g/V_l . The same correction factors are also applied to the thermodynamic reaction functions measured for each *strain.*

10. **Calculate Boltzmann Distribution Constants** Assume that the probability distribution elementary modes can be estimated by one of the two formulas

$$p_j = \exp\left(-\frac{\hat{\xi} \Delta s_j}{bQk} + c\right) \qquad p_j = \exp\left(-\frac{\hat{\xi}\, \Delta g_j}{bQkT} + c\right)$$

Where $\varDelta s_j$ and $\varDelta g_j$ are the thermodynamics reaction functions for each mode calculated under actual working conditions. Use the technique of Lagrange multipliers to enforce the constraints of $\sum p_j = 1$ and $\sum p_j \varDelta s_j = \overline{\Delta S_R}$ (or $\sum p_j \varDelta g_j = \overline{\Delta G_R}$)(where $\overline{\Delta S_R}$ and $\overline{\Delta G_R}$ are computed using the *experimental* stoichiometric equation that is specific to each strain) in order to determine the values of the constants *b* and c.

11. **Model Estimation of Fluxes** Once individual p_i are determined, estimate mean fluxes according to the equation $\bar{f_z} = \sum p_i f_{z,i}$
